# Transport and Recovery of Turbot (*Scophthalmus maximus*) Sedated with MS-222 and Eugenol: Effects on Intermediary Metabolism and Osmoregulation

**DOI:** 10.3390/ani11082228

**Published:** 2021-07-29

**Authors:** Jie Cao, Qi Wang, Weiqiang Qiu, Jun Mei, Jing Xie

**Affiliations:** 1 College of Food Science and Technology, Shanghai Ocean University, Shanghai 201306, China; m190300743@st.shou.edu.cn (J.C.); m190310920@st.shou.edu.cn (Q.W.); wqqiu@shou.edu.cn (W.Q.); 2 National Experimental Teaching Demonstration Center for Food Science and Engineering, Shanghai Ocean University, Shanghai 201306, China; 3 Shanghai Engineering Research Center of Aquatic Product Processing and Preservation, Shanghai 201306, China; 4 Shanghai Professional Technology Service Platform on Cold Chain Equipment Performance and Energy Saving Evaluation, Shanghai 201306, China

**Keywords:** anesthesia, turbot, waterless transport, physiological indicators, biochemical indicators

## Abstract

**Simple Summary:**

The waterless transportation of live fish is considered to be a green and economic solution to achieve a high survival rate. However, the effects of cold domestication and the environment after leaving the water are prone to cause stress in fish. In this experiment, the turbots were anesthetized with 40 mg/L MS-222 and 20 mg/L eugenol solution. Then, turbots were cooled to dormancy, followed by waterless transport for 18 h. The results of blood physiological indices indicated that the MS-222 and eugenol could reduce the stress on turbot during cooling and simulated waterless transport.

**Abstract:**

This study focused on the anesthetic waterless keep-alive transport technique for turbot. MS-222 and eugenol were used to anesthetize turbot and then the waterless keep-alive transport was conducted. The blood physiological changes and flesh quality changes of turbot were evaluated after cooling and during the simulated waterless transport. The results show that the temperature lowered from 13 to 2 °C, resulting in a decrease in moisture, fat and protein contents of all samples. Compared to the control turbot, the turbots treated with MS-222 and eugenol presented higher pH and glycogen content. During the simulated waterless transport, the pH, ATP and glycogen contents in MS-222- and eugenol-treated turbots decreased and the IMP and lactate levels increased. For the blood biochemical indices, blood glucose, cortisol and urea nitrogen increased with the increase in transport time in MS-222- and eugenol-treated turbots. At sampling time, the changes in blood physiological indices were significantly higher in the control samples than those in the MS-222- and eugenol-treated samples. The results indicate that the turbot samples treated with MS-222 or eugenol could reduce stress during cooling and simulated waterless transport.

## 1. Introduction

Turbot (*Scophthalmus maximus*) is one of the most important economic fish in China and its output in 2019 exceeded 116,000 tons (China Fishery Statistical Yearbook, 2020). Most turbots are sold in the domestic market as fresh chilled products rather than as live fish. However, traditional live-water transportation techniques for turbot are usually accompanied by high costs and high rates of loss over long distances, thus significantly limiting the development of the live fish market industry [1]. It is important to explore new transportation techniques to improve the transportation of live turbot by reducing costs, increasing survival rates and extending transit times.

Waterless transportation of live fish is considered as a green and economical solution to achieve high survival rates and quantity [2,3]. Several studies have shown that waterless transport methods have been applied to the live transport of some cold-water fish species, such as sturgeon farmed in China and rainbow trout fingerlings [2]. The key to successful transportation of waterless fish is the cold-induced dormancy. However, cold acclimation could cause oxidative stress, affect the immune system and even cause tissue damage, which reduces the viability of acclimated fish [4]. The use of slow physical cooling and anesthetic treatment could reduce the oxidative stress of the fish in the early stage of keep-alive transportation and the stress response after leaving the water environment, avoiding the fish’s struggle, reducing the level of metabolism and prolonging the waterless transportation time.

Fish anesthetics have been widely used in water transportation of live fish. MS-222 and eugenol are the most widely used. Eugenol (4-butallyl-2-methoxyphenol) is the main component of clove oil. It has the advantages of good practicability and low price, so it has been widely used. Existing studies show that eugenol has been applied in the anesthetic transportation of freshwater angelfish, juvenile (*Lophiosilurus alexandri*) and Nile tilapia [5,6,7]. Tricaine methane-sulfonate, or MS-222, it is an inhalation anesthetic, usually used in fish [8]. MS-222 has low drug concentration, fast anesthesia, fast recovery and no toxic side effects. It is currently the only anesthetic used by the Food and Drug Administration (USFDA) for food fish [9]. At present, MS-222 has been used in the anesthesia and transportation research of Largemouth Bass, pikeperch (*Sander lucioperca*), lumpfish (*Cyclopterus lumpus*) and Far Eastern Catfish (*Silurus asotus*), and so on [10,11,12,13]. Feng G et al. and Palić D et al. also studied the effects of MS-222 and eugenol on blood biochemical parameters of juvenile Siberian sturgeon (*Acipenser baerii*) and on the cellular function of fathead minnows (*Pimephales promelas*) [14,15]. In addition, Teles M et al. studied the effect of eugenol and MS-222 on the oxidative stress state of gilthead sea bream (*Sparus aurata* L.) during anesthetized transport and after recovery [16]. In this experiment, eugenol and MS-222 were used to anesthetize turbot, and then proceed to waterless transportation. The purpose of this study was to provide a theoretical basis for the application of waterless transport methods for turbot to help keep the fish alive and maintain its vitality and quality.

## 2. Materials and Methods

### 2.1. Preparation of Turbot

The experimental protocol was approved by the Institutional Animal Care and Use Committee of Shanghai Ocean University (SHOU-DW-2021-066). A total of 100 live cultured turbot (weight 600 ± 50 g) were purchased from the local market in Luchao port town (Shanghai, China) and then transported to the laboratory in a truck equipped with holding tanks. The fish were kept in prepared polyethylene-based tanks (2.4 × 1.7 × 0.6 m^3^) for 2 days prior to the experiment to acclimatize to the experimental environment. The average water temperature was 13 °C, the salinity of seawater was 30‰, the average pH was 7.5 and the average dissolved oxygen was 6.0 mg∙L^−1^.

MS-222 and the corresponding weight of sodium bicarbonate were dissolved in seawater at concentrations of 40 mg/L. Considering the hydrophobicity of eugenol, the anesthetic solution was prepared by dilution in 95% ethanol at a ratio of 1:9 to make a stock solution (100 g/L) for later use [17]. Then, the eugenol solution was dissolved in seawater to prepare 20 mg/L eugenol solution. These concentrations were determined in pre-tests.

The turbot was divided into 3 groups: (a) M-222 treatment: 30 healthy turbots treated with 40 mg/L MS-222 solution; (b) Eugenol treatment: 30 healthy turbots treated with 20 mg/L eugenol solution; and (c) Control treatment: the turbot was transported without anesthesia treatment. After turbots were anesthetized, the water temperature dropped from 13 to 2 °C at a rate of 1 °C per hour. After cooling to 2 °C, the fish were confirmed to be in a dormant state and their fins and gills were observed to be almost immobile [4]. The dormant turbots were gently put into a plastic bag that was then filled with oxygen. Then, the turbots were put into an insulated box and the waterless transport was simulated in a vibration conveyor under 100 rpm at 2 °C for 18 h.

### 2.2. Measurements and Analytical Procedures

#### 2.2.1. Sample Collection

Three turbot samples were randomly selected from each group and analyzed at 0, 6, 12 and 18 h during simulated waterless transport and after 48 h of recovery in water, respectively. A 2 mL disposable syringe was used to draw blood from the tail vein without adding anticoagulant. Blood was transferred to 2.0 mL centrifuge tubes and let stand in a refrigerator at 4 °C for 2 h. Then, it was centrifuged at 3000 rpm for 15 min. The serum was collected in a 1.5 mL centrifuge tube and stored at −20 °C until analysis [18]. Flesh tissues from the back and abdomen of each fish were collected for biochemical analysis.

#### 2.2.2. Chemical Composition of Flesh

In the experiment, the water, crude fat and crude protein contents of turbot muscle were determined by determined according to the national standard method [19].

#### 2.2.3. Flesh pH

A pH probe (Testo 205, Detu Instruments International Trade Co., Shanghai, China) was inserted to measure flesh pH after slaughter. The flesh pH was measured at three different parts of the fish body; the upper part of the fish fillet, the back part of the back flesh and the abdominal flesh. The flesh pH was calculated as the average of three measurements.

#### 2.2.4. Biochemical Analysis of Flesh Samples

Determination of lactic acid and glycogen in turbot flesh was achieved by measuring with lactic acid and glycogen (Nanjing Jiancheng Institute of Bioengineering, Nanjing, China) and reading with a UV-2100 UV-Visible Spectrophotometer (Unico Instruments Co., Ltd., Franksville, WI, USA).

#### 2.2.5. Determination of Nucleotides

Nucleotide extracts were prepared according to the method of Fang et al. [20]. ATP-related compounds, including adenosine triphosphate (ATP), inosine monophosphate (IMP) and adenosine monophosphate (AMP), were analyzed while using HPLC (Waters 2695, Milford, CT, USA), equipped with a VP-CDS C18 column (150 × 46 mm).

#### 2.2.6. Determination of Serum Biochemical Indicators

The biochemical indices of serum, including glucose (GLU) and blood urea nitrogen (BUN), were determined by using commercial assay kits (Nanjing Jiancheng Institute of Bioengineering, Nanjing, China) according to the manufacturer’s instructions. Serum cortisol was determined by means of an enzyme-linked immunoassay according to the Cortisol ELISA Kit manufacturer’s instructions (Shanghai Fanke Industrial Co., Ltd., Shanghai, China).

#### 2.2.7. Statistical Analysis

Data were expressed as the mean ± SD and the one-way analysis of variance (ANOVA) procedure (International Business Machines Corporation, Armonk, NY, USA) followed by Duncan’s multiple range tests was adopted to determine the significant difference (*p* < 0.05) between treatments.

## 3. Results

### 3.1. Chemical Composition of Flesh

As shown in Table 1, the moisture and protein contents of turbot in each treatment group were significantly reduced when the temperature was lowered from 13 to 2 °C dormant temperature (*p* < 0.05), in which the water and protein content of turbot in the control group decreased by 0.78% and 0.57%, while the water and protein content of turbot in the MS-222-treated group decreased by 0.45% and 0.42%, and the water and protein content of turbot in the eugenol-treated group decreased by 0.47% and 0.40%. After 18 h of simulated transport, the moisture, fat and protein contents of turbot flesh in each treatment group decreased significantly (*p* < 0.05) compared with the turbot flesh at 0 h. Among them, the moisture and protein contents of turbot flesh in the CK sample decreased by 0.60% and 0.46% at the end of simulated transport, while the moisture and protein content of turbot in the MS-222-treated group decreased by 0.57% and 0.42%, and the moisture and protein content of turbot in the eugenol-treated group decreased by 0.47% and 0.34%. The results show that low-temperature stress also had as great effect as that of transport stress on flesh quality. The moisture, fat and protein contents of turbot in each treatment group were restored to their initial levels after 48 h of resuscitation.

### 3.2. Flesh Biochemical Indicators

**Figure 1:**
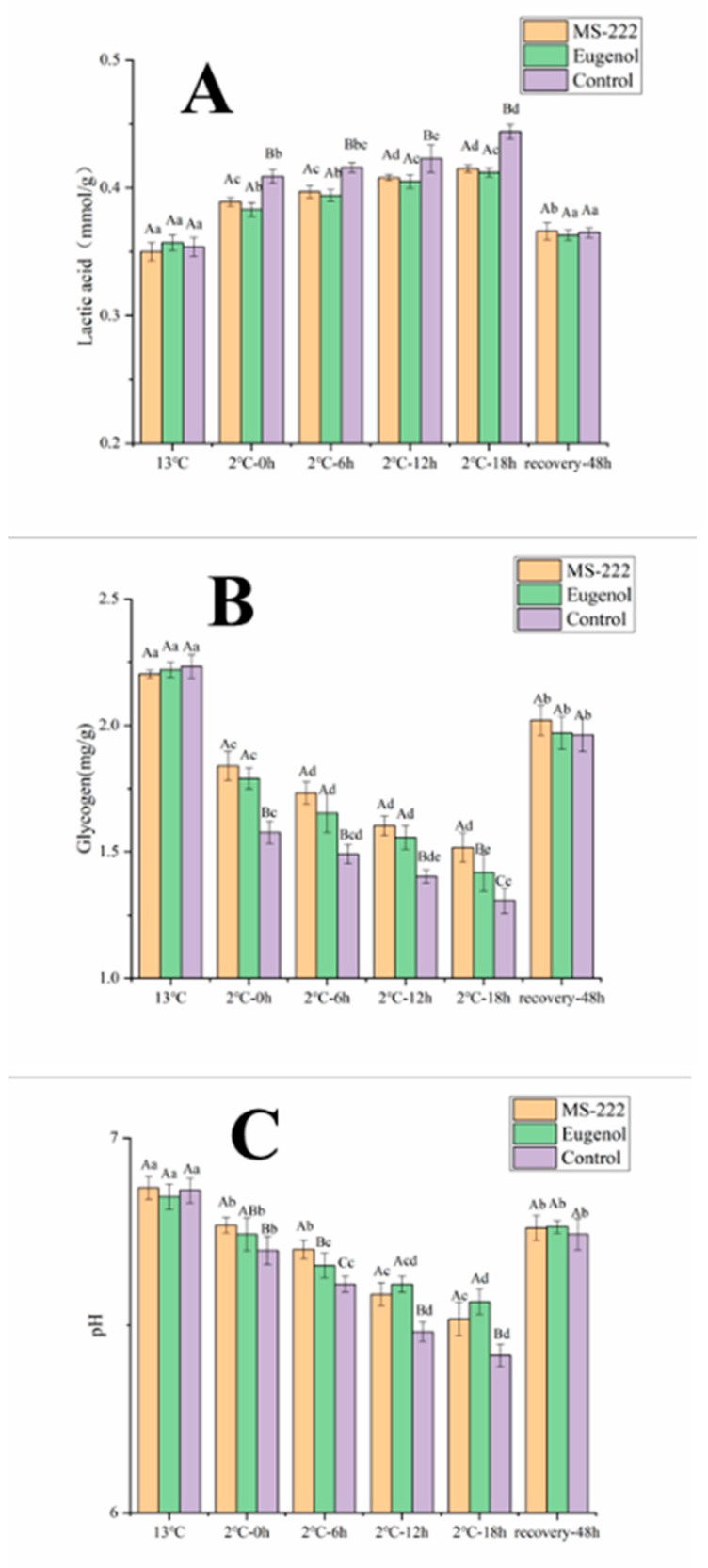
Flesh glycogen (**A**), lactic acid (**B**) and pH (**C**) changes of turbot affected by low temperature transport stress. Different capital letters indicate significant differences in means between different treatment groups. Different lowercase letters indicate significant differences in means between the same treatment groups (*p* < 0.05).

The changes in lactic acid, glycogen contents and pH value of turbot during low-temperature waterless transport are shown in Figure 1. The flesh glycogen contents of turbot in all samples decreased significantly (*p* < 0.05) when the water temperature decreased from 13 to 2 °C. Additionally, the pH and glycogen content of turbot in the MS-222- and eugenol-treated groups were significantly higher than those in the control group. After 18 h of simulated transport, the turbots in control group showed a 21% increase in lactic acid content and a 17% decrease in glycogen content; the turbots in the MS-222 treated group showed a 13% increase in lactic acid content and a 12% decrease in glycogen content, and the turbots in the eugenol-treated turbot group showed a 12% increase in lactic acid content and a 14% decrease in glycogen content. After the simulated transport, the flesh pH of turbot decreased from 6.82 to 6.42 in the control group, from 6.86 to 6.52 in the MS-222-treated group and from 6.84 to 6.56 in the eugenol-treated group. The lactic acid, glycogen contents and pH value of all turbot samples returned to the initial level after 48 h of resuscitation.

### 3.3. Changes in Blood Glucose and Flesh ATP in Turbot during Simulated Transport

**Figure 2:**
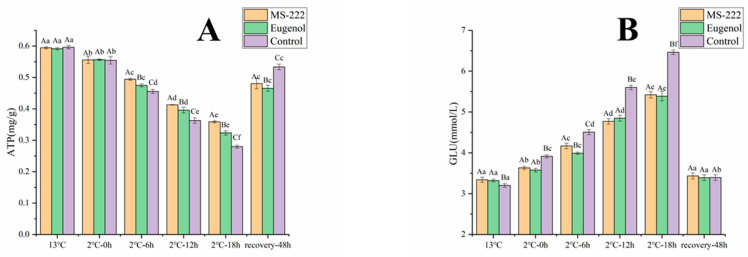
ATP (**A**) and blood glucose (**B**) changes in turbot affected by low-temperature transport stress. Different capital letters indicate significant differences in means between different treatment groups. Different lowercase letters indicate significant differences in means between the same treatment groups (*p* < 0.05).

The flesh ATP content of turbot remained at a high level after cooling and at 0 h of transport. There was no significant change in the flesh ATP content of turbot in each treatment group after the end of cold domestication. The results show that the ATP contents in turbot flesh were significantly lower at the end of transport compared with those at 0 h. The flesh ATP content of turbot in the control group was significantly lower than that of turbot in the MS-222- and eugenol-treated groups (*p* < 0.05). Glucose levels in turbot blood increased with transport time and the CK sample had a more pronounced increase (Figure 2B). At each sampling time point, glucose levels were significantly higher in control turbot than in fish from the MS-222 and eugenol treatment groups (*p* < 0.05).

### 3.4. Changes of Cortisol and BUN in Turbot during Waterless Transport

**Figure 3:**
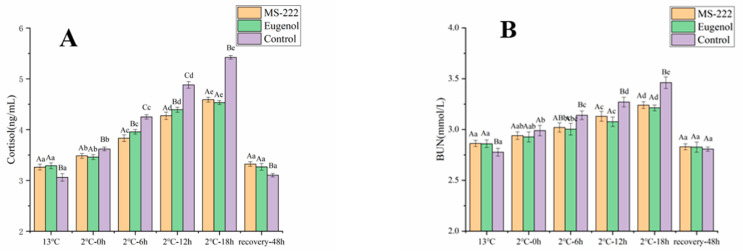
Cortisol (**A**) and BUN (**B**) changes in turbot affected by low-temperature transport stress. Different capital letters indicate significant differences in means between different treatment groups. Different lowercase letters indicate significant differences in means between the same treatment groups (*p* < 0.05).

Serum cortisol levels were significantly elevated at the end of transport in all turbot treatment groups, with a 50% increase in the control group, a 32% increase in the MS-222-treated group and a 30% increase in the eugenol-treated group. Cortisol levels gradually returned to initial levels after 48 h of resuscitation (Figure 3). These results suggest that MS-222 and eugenol preanesthetic treatments were able to inhibit the increase in blood cortisol levels compared with the control group.

Blood urea nitrogen concentrations changed slowly in the MS-222- and eugenol-treated turbot samples compared with the CK samples during the simulated transport. In contrast, blood urea nitrogen concentrations increased significantly in the control group, increasing to 3.27 and 3.46 mmol/L at the 12th and 18th h, respectively. Blood urea nitrogen levels returned to the initial levels in all samples after 48 h of resuscitation.

### 3.5. Changes of Nucleotides in Turbot

The changes in flesh flavor nucleotide content of turbot during waterless transport are shown in Table 2. The IMP value gradually increased and the AMP value decreased during simulated transport. At the end of transport, the increase in IMP of turbot flesh in the control group was 28.47% and TAV increased to 14.79; the increase in IMP of turbot flesh in the MS-222-treated group was 18.06% and TAV increased to 13.99; and the increase in IMP of turbot flesh in the eugenol-treated group was 12.44% and TAV increased to 13.96. However, the AMP values decreased from hour to hour and then increased to the end of transport. The flesh AMP in CK, MS-222- and eugenol-treated samples decreased by 14.72%, 0.6% and 4.2%, respectively.

## 4. Discussion

Changes in temperature and transportation put tremendous pressure on fish [21], which led to the changes in the chemical composition of turbot flesh. The turbot secretes mucus to protect themselves during the waterless transportation, resulting in water loss. Mohamed et al. suggested that the reduction in the moisture content of fish after transport could be the result of metabolic disorders and abnormal enzyme functions [2]. Jrpeland et al. found that the water content of stressed *Gadus morhua* L. was significantly lower compared to unstressed fish [22].

During the waterless transportation of turbot, the content of protein and fat decreased significantly. It is speculated that the reasons for the decrease in the content of energy-supplying substances such as protein and fat in the fish are on the one hand, because the fish is in a waterless low-temperature state during transportation, in order to provide nutrients to the organism and keep it alive, some of the nutrients in the fish meat were degraded, resulting in the denaturation of flesh protein [23]. On the other hand, the unfavorable environmental factors, such as vibration during transportation, continue to stimulate the fish body and consume energy, thereby promoting the decomposition of energy supply material. In this experiment, it was observed that there was no significant difference in flesh qualitative parameters between the anesthetized group turbot and the control group turbot flesh qualitative parameters. After 48 h of resuscitation, the basic nutrients of the fish’s flesh could be restored to the level of the control group, and the consumption of the fish weight by simulated waterless transportation was negligible.

From the current research, under the stress of low temperature and transport, turbots maintain the energy requirements of the organism by catabolizing flesh glycogen. This may be due to the energy requirements during transportation. Once under stressful conditions, flesh glycogen reserves are mobilized to provide energy [24]. The decrease in oxygen during transport leads to an increase in anaerobic metabolism and the conversion of glycogen to lactate. According to Moraes et al. [25], reduced oxygen level is a common source of stress in fish, leading to a large export of lactate into the plasma. The increased lactate content in fish suggests that fish are unable to maintain initial internal homeostasis. Flesh pH is an important meat quality parameter and the accumulation of lactic acid in the flesh during transport leads to a decrease in flesh pH [26], which is consistent with the results obtained in this study. The decrease in flesh pH will reduce the quality of the flesh, through means such as loss of water retention capacity, and change the texture of the flesh [23]. The rate of decrease in glycogen and the rate of increase in lactic acid in the flesh of turbot in MS-222- and eugenol-treated groups were smaller than those in the control group during waterless transportation, which was because the fish were anesthetized, the metabolic rate was reduced and less lactic acid was accumulated in the flesh tissue, so the decrease spokes of glycogen and pH in the anesthetized turbot flesh were lower than those in the control group.

Adenosine triphosphate (ATP) provides the energy required to maintain normal life activities. Changes in the levels of ATP and its metabolites can indicate changes in the carnal energy of fish [27]. When the ambient temperature decreases, the main adaptive change that occurs in the organism is an increase in blood glucose levels. During cold stress conditions or prolonged exposure to low temperatures, the body converts large amounts of glucose into ATP to provide energy [28]. In the present study, blood glucose levels increased with transport time, indicating that that under waterless conditions, the fish body needs to release more glucose to maintain normal metabolism as the amount of respirable oxygen decreases It was found that the muscle ATP content of turbot in MS-222- and eugenol-treated groups was significantly lower than that of turbot in the control group at the end of transport, while the blood glucose level was significantly lower than that of the control group. This result suggests that the anesthetics can reduce the energy metabolism of the fish during transport, which is also similar to the findings of Eduardo et al. [29].

Serum cortisol is one of the most important indicators of stress levels in fish [30]. Under cold and stressful conditions, serum cortisol levels in fish increase significantly [27]. In addition, blood urea nitrogen (BUN), which is formed in the liver and excreted through the kidneys and is an important indicator of kidney function [31], also increases. Under stressful conditions, increased levels of cortisol and urea nitrogen facilitate the regulation of osmotic pressure and the maintenance of vital characteristics in bony fish [32]. Barton et al. showed that transport causes stress and increases serum cortisol levels in live fish [33]. The conditions of the external environment will activate the HPI system in fish, leading to the production of large amounts of cortisol hormones to regulate the ion permeability of cells and maintain physiological homeostasis [34]. In this study, cortisol levels in MS-222-treated and eugenol-treated turbots were significantly lower than those in the control group after cooling and transport. This is similar to the results obtained by Jerez-Cepa et al., suggesting that anesthetics can reduce the effects of transport stress [16]. The result of the study shows that anesthetized preservation of transported turbot could reduce the increase in cortisol and contribute to the survival of the fish.

ATP and its related compounds have taste characteristics and are the main source of taste in fish. IMP and AMP were the two main taste nucleotides in turbot during simulated transport. The TAV values of IMP in turbot flesh were greater than 10, indicating that IMP was the main source of taste in turbot flesh at 2 °C–0 h, the IMP content of turbot in each treatment group was significantly lower than that of fish in the 13 °C treatment group, which was a temporary effect of the cooling operation on taste presenting substances. With the extension of transport time, the breakdown of ATP in turbot flesh led to the accumulation of IMP, and the content of IMP increased significantly, which is the same as the results of Wu Bo et al., who found a significant increase in IMP content in grouper flesh at the end of transport [35]. With the extension of transportation time, the AMP content of turbot first decreased and then increased, and after 18 h, AMP was still at a high level, indicating that the low-temperature anhydrous transportation operation could increase the fresh flavor-presenting substances of turbot and improve the flavor of fish flesh. It was observed in this experiment that the level of IMP in the flesh of turbot in the control group was significantly higher than that in the MS-222- and eugenol-treated groups during anhydrous transport, due to the fact that the anesthetic manipulation reduced the energy metabolism of the fish and slowed down the degradation of ATP in the flesh. Thus, the IMP in the flesh of turbot in the treated group showed higher levels.

## 5. Conclusions

The results of the study show that after the pre-treatment with MS-222 and eugenol anesthesia, the large amount of energy consumed in the cooling process could be avoided, the cost savings could be substantial and the convenience, speed and time saving further met the requirements of commercial transportation of turbot. Because turbot has the function of skin respiration, in this kind of fish waterless keep-alive transport makes full use of the function of skin respiration, in maintaining the environmental requirements of the fish needed to maintain a certain level of humidity and oxygen, to prevent fish tissue dehydration and hypoxia. In the process of waterless keep-alive transport, water, crude fat and protein content of fish flesh decreased, glycogen content decreased, lactic acid content increased and flesh pH value decreased; blood glucose, cortisol and urea nitrogen content in blood increased significantly in the process of waterless keep-alive transport.

## Figures and Tables

**Table 1:** The effect of low-temperature waterless transport stress on the chemical composition of turbot flesh.

Indicators/Transport	Samples	13 °C	2 °C—0 h	2 °C—6 h	2 °C—12 h	12 °C—18 h	Recovery—48 h
Water content (%)	Control	78.89 ± 0.26 ^Aa^	78.11 ± 0.10 ^Ab^	77.87 ± 0.11 ^Ac^	77.63 ± 0.06 ^Ad^	77.51 ± 0.08 ^Ad^	78.80 ± 0.12 ^Aa^
	MS-222	78.77 ± 0.19 ^Aa^	78.32 ± 0.08 ^Bb^	78.11 ± 0.12 ^ABc^	77.93 ± 0.13 ^Bd^	77.75 ± 0.05 ^Bd^	78.67 ± 0.09 ^Aa^
	Eugenol	78.74 ± 0.33 ^Aa^	78.27 ± 0.11 ^ABb^	78.10 ± 0.05 ^Bc^	77.95 ± 0.07 ^Bd^	77.80 ± 0.10 ^Bd^	78.66 ± 0.15 ^Aa^
Crude fat (%)	Control	1.44 ± 0.06 ^Aa^	1.24 ± 0.04 ^Ab^	1.17 ± 0.03 ^Abc^	1.10 ± 0.04 ^Acd^	1.04 ± 0.05 ^Ad^	1.39 ± 0.08 ^Aa^
	MS-222	1.47 ± 0.05 ^Aa^	1.32 ± 0.07 ^Bb^	1.24 ± 0.05 ^Abc^	1.16 ± 0.03 ^Acd^	1.10 ± 0.06 ^Ad^	1.41 ± 0.07 ^Aa^
	Eugenol	1.40 ± 0.03 ^Aa^	1.28 ± 0.05 ^ABb^	1.22 ± 0.04 ^Ab^	1.18 ± 0.05 ^Abc^	1.12 ± 0.05 ^Ac^	1.37 ± 0.06 ^Aa^
Crude protein (%)	Control	18.65 ± 0.11 ^Aa^	18.08 ± 0.10 ^Ab^	17.95 ± 0.05 ^Ab^	17.83 ± 0.16 ^Abc^	17.62 ± 0.10 ^Ac^	18.51 ± 0.09 ^Aa^
	MS-222	18.61 ± 0.07 ^Aa^	18.19 ± 0.09 ^ABb^	18.06 ± 0.14 ^ABb^	17.98 ± 0.11 ^Ab^	17.89 ± 0.14 ^ABb^	18.60 ± 0.11 ^Aa^
	Eugenol	18.66 ± 0.09 ^Aa^	18.26 ± 0.0.05 ^Bb^	18.13 ± 0.08 ^Bb^	18.00 ± 0.10 ^Abc^	17.92 ± 0.07 ^Bc^	18.69 ± 0.13 ^Aa^

Different capital letters indicate significant differences in means between different treatment groups. Different lowercase letters indicate significant differences in means between the same treatment groups (*p* < 0.05).

**Table 2:** Changes in nucleotides of turbot during waterless transport.

Transport (h)	Sample	IMP (mg/100 g)	TAV	AMP (mg/100 g)	TAV
13 °C	Control	319.44 ± 11.55 ^Ab^	12.78	24.73 ± 0.71 ^Aa^	0.49
	MS-222	312.45 ± 6.11 ^Ab^	12.50	20.96 ± 0.76 ^Ba^	0.42
	Eugenol	310.37 ± 5.20 ^Ab^	12.41	21.62 ± 1.09 ^Ba^	0.43
2 °C—0 h	Control	287.81 ± 7.29 ^Aa^	11.51	19.41 ± 1.14 ^Ab^	0.39
	MS-222	296.17 ± 5.16 ^Aa^	11.85	18.23 ± 0.97 ^Ab^	0.36
	Eugenol	292.50 ± 5.65 ^Aa^	11.70	18.96 ± 1.63 ^Abc^	0.38
2 °C—6 h	Control	343.39 ± 7.86 ^Ac^	13.74	16.65 ± 0.80 ^Ac^	0.33
	MS-222	325.91 ± 5.25 ^Bc^	13.04	15.99 ± 0.63 ^Ac^	0.32
	Eugenol	325.63 ± 3.31 ^Bc^	13.03	16.74 ± 1.41 ^Acd^	0.33
2 °C—12 h	Control	356.18 ± 6.04 ^Acd^	14.25	13.81 ± 0.47 ^Bd^	0.28
	MS-222	340.62 ± 2.36 ^Bd^	13.62	14.55 ± 0.48 ^ABc^	0.29
	Eugenol	341.49 ± 5.69 ^Bd^	13.66	15.41 ± 1.21 ^Ad^	0.31
2 °C—18 h	Control	369.76 ± 7.22 ^Ad^	14.79	16.92 ± 1.39 ^Bc^	0.34
	MS-222	349.67 ± 1.44 ^Be^	13.99	18.11 ± 1.19 ^Ab^	0.36
	Eugenol	348.97 ± 6.69 ^Bd^	13.96	18.2 ± 0.23 ^Abc^	0.36
Recovery—48 h	Control	320.00 ± 13.62 ^Ab^	12.80	23.24 ± 0.56 ^Ba^	0.46
	MS-222	322.78 ± 3.80 ^Ac^	12.91	20.64 ± 0.83 ^Aa^	0.41
	Eugenol	314.93 ± 4.14 ^Ab^	12.60	20.91 ± 1.87 ^Aab^	0.42

Different capital letters indicate significant differences in means between different treatment groups. Different lowercase letters indicate significant differences in means between the same treatment groups (*p* < 0.05). TAV: Taste activity value. (IMP: 25 mg/100 g, AMP: 50 mg/100 g). IMP: inosine monophosphate. AMP: Adenosine monophosphate.

## Data Availability

All data, models, and code generated or used during the study appear in the submitted article.
